# Impact of atmospheric NO_2_ on pediatric asthma visits in Jinan: effect modification by season and apparent temperature

**DOI:** 10.3389/fpubh.2026.1793681

**Published:** 2026-05-08

**Authors:** Xiaoling Wei, Huiyun Chang, Min Xue, Beilei Wang, Ming Liu, Liheng Wang, Liangliang Cui, Xiang Ma

**Affiliations:** 1 Jinan Children's Hospital, Jinan, China; 2 Jinan Municipal Center for Disease Control and Prevention, Jinan, China; 3 Jinan Mental Health Center, Jinan, China

**Keywords:** apparent temperature, pediatric asthma visits, nitrogen dioxide, seasons, time-stratified case-crossover study

## Abstract

**Background:**

Childhood asthma shows seasonal/Solar terms (STs) patterns, affected by air pollution (notably Nitrogen Dioxide-NO_2,_ most sensitive to pediatric asthma) and meteorological factors. Apparent temperature (AT) integrates temperature and other meteorological indices. However, joint effects of multiple factors remain unknown.

**Methods:**

Daily pediatric asthma visits of Children’s Hospital Affiliated to Shandong University, meteorological factors and air pollution concentrations were collected from January 1, 2018 to December 31, 2022. Time-stratified case crossover and conditional logistic regression models were utilized to estimate the odds ratios (*OR*s) and 95% confidence intervals (*CI*s) of effects of asthma visits.

**Results:**

29,906 asthma visits were recorded with age range of 0.8–19 years. Median NO₂ concentration of 33 μg/m^3^ and 14 visits recorded per day. NO_2_ exposure increased risk of asthma visits in children [*OR* = 1.005 (95%*CI*: 1.003, 1.007), Lag05]. Moreover, the association between NO₂ exposure and the risk of pediatric asthma visits exhibited an approximately parabolic shape, with the nadir of risk at a NO₂ concentration of 33 μg/m^3^. Stratified analysis of demographic characteristics revealed higher effect estimate in females [*OR* = 1.006 (95%*CI*: 1.002, 1.009), Lag05] and the strongest effects of age in children and adolescents aged 10–19 years [*OR* = 1.005 (95%*CI*: 1.001, 1.009), Lag3]. A higher effect estimate was observed during the summer–autumn seasons [*OR* = 1.006 (95%*CI*: 1.002, 1.009), Lag02]. The interaction between atmospheric NO_2_ and low (AT) were significant among total visits [*OR* = 1.002 (95%*CI*: 1.001, 1.004)], with a moderate AT used as a reference.

**Conclusion:**

NO_2_ considerably increased pediatric asthma visits, seasons and AT having modifying effects. These findings highlight the urgent need for increased awareness and action from relevant agencies.

## 1 Introduction

Asthma poses a serious global health problem, it affects approximately 300 million people globally and causes around 1,000 deaths per day. It is the world’s second leading cause of death from chronic respiratory diseases, affecting all ages and causing severe disability and death globally, particularly in low- and middle-income countries (1, 2). Asthma in children disrupts people’s work, education and family life; without thorough treatment, this condition can continue into adulthood, with severe cases posing life-threatening risks, which substantially affects children’s physical and mental health and increases the financial burden on families and society (3). Therefore, asthma prevention and control efforts are critically important for both the families of affected children and society as a whole. Meanwhile, multiple factors, such as exercise, allergens, irritants, climate change, air pollution and viral respiratory infections, can exacerbate and prolong asthma condition. Crucially, environmental exposures represent a major modifiable cause of this burden, influencing both disease exacerbation and treatment responsiveness. Addressing these environmental risks is therefore a vital component of effective asthma management. Outdoor air pollution can trigger asthma symptoms and exacerbations, especially nitrogen dioxide (NO_2_) is more sensitive (4). In the context of climate change, the effect of temperature variability on respiratory health is becoming increasingly evident (5). Climate change can trigger and exacerbate asthma attacks (6). Moreover, the occurrence of asthma in children exhibited seasonal and Solar terms (STs) patterns (7, 8). The 24 STs, a unique traditional Chinese calendar, were established by ancient Chinese scholars over 2000 years ago (9). The 24 STs are guiding people’s agricultural pursuits, daily routines, and healthcare practices from antiquity to the present (10).

A few studies have focused on the pediatric population, which has unique physiological characteristics and sensitivities. Although some research have explored the relationship between weather and asthma outpatient visits, they only demonstrated the positive or negative associations of these variables with the incidence rate of pediatric asthma (6). However, the influencing factors are complex and may involve unobserved confounders, such as interactions between variables. Therefore we aimed to explore the relationship between atmospheric NO₂, seasons, apparent temperature (AT) and pediatric asthma visits in children from a combined perspective. Through this approach, we gained more in-depth insights into the complex relationship between weather-related factors and asthma and formulated targeted recommendations for the prevention and control of pediatric asthma exacerbations. The results of this work will fill the gaps of previous research and contribute to the improvement of strategies on pediatric asthma management.

## 2 Materials and methods

### 2.1 Study area

Jinan City, the capital of Shandong Province, is situated between 36°02′ –37°54′N latitude and 116°21′–117°93′E longitude. It spans a total area of 10,244 km^2^ and is divided into 10 administrative districts, which house approximately 9 million people (11). The city experiences a classic temperate monsoon climate characterized by chilly winters, hot summers and distinct seasonal changes. Jinan City has undergone swift industrialization and urbanization processes. This area serves as a pivotal link between two prominent economic zones, namely, the Beijing-Tianjin-Hebei Regions and the Yangtze River Delta, which suffer from substantial air pollution. In particular, NO_2_ is a pollutant that shows a slower rate of improvement after the implementation of air pollution control actions (12).

### 2.2 Study population

This work included children and adolescents with asthma who visited the Children’s Hospital affiliated to Shandong University (CHSU) from January 1, 2018 to December 31, 2022. This Grade 3A children’s hospital in Jinan City is a national standardized outpatient demonstration center for children’s asthma, which has a certain degree of representation. This study included 29,906 records of asthma visits from Children’s Asthma Cohort Study database created by the Department of Respiratory and Asthma Centre in CHSU. The databases mainly comprised basic patient information (gender and age), type of visit (outpatient and inpatient), date of visit and diagnosis. The diagnosis of asthma was based on the guidelines for the diagnosis and treatment of bronchial asthma in children (2016) (13). Specifically, the diagnostic coding was strictly implemented in accordance with International Classification of Diseases, 10th revision (ICD-10 codes: J45-J46) with non-compliant cases excluded. Duplicate records of patients admitted within 24 h after outpatient or ED visits were classified as inpatient cases for calculation, with their outpatient records excluded simultaneously.

### 2.3 Data collection on air pollutions and meteorological variables

The daily mean concentrations of NO_2_ and other pollutants (PM_2.5_, PM_10_, SO_2_, CO and O_3_-8h), were obtained from the Jinan Ecological Environmental Protection Bureau (http://jnepb.jinan.gov.cn/). Meteorological data, including daily mean temperature (°C), relative humidity (RH, %), wind speed (WS, m/s) and pressure (hPa), were obtained from the China Meteorological Science Data Sharing Service (http://data.cma.gov.cn/). Meanwhile, we performed quality control on the collected data; details are provided in the Supplementary materials.

### 2.4 Calculation of apparent temperature

Referring to previous literature, the meteorological factor indicators, including daily mean temperature, RH and WS, were used to calculate AT. The specific formula is as follows (14, 15):


AT=Tmean+0.33e−0.70WS−4.00 (1)

e=(RH/100)×6.105×exp[(17.27×Tmean)/(237.7+Tmean)] (2)

In Equations 1, 2, AT denotes the daily apparent temperature, press refers to the daily mean pressure; WS indicates the daily mean wind speed; RH is daily mean relative humidity; Tmean is the daily mean temperature.

AT is defined as follows: An extremely low AT is less than or equal to the 5th percentile of AT; a low AT is between the 5th and 25th percentiles of the AT; moderate AT is between the 25th and 75th percentile of the AT; high AT is between the 75th and 95th percentile of the AT; extremely high AT is more than or equal to the 95th percentile of AT.

### 2.5 Statistical analysis

Our analysis involved a three-stage process. We initially performed descriptive analysis on the collected data, using indicators, such as minimum (Min), maximum (Max), median (M), first quartile (*P*_25_) and third quartile (*P*_75_). Secondly, we tested the Spearman correlation between air pollutants and meteorological factors. Then, we conducted a time-stratified case-crossover study using a conditional logistic regression model to examine the association between NO_2_ exposure and asthma visits of children and adolescents. In consideration of the time-stratified case-crossover study design, the study period was divided into time strata, with the case phase and control phase occurring within the same stratum. For instance, if an asthma visit occurred on January 17, 2018 (a Wednesday), other Wednesdays in January 2018 were considered control days. Furthermore, given the confounding factors, AT and holiday effects were incorporated into the regression model as covariates. Given the lag effect of NO_2_ exposure on human health, with the primary exposure-effect window identified between Lag3 to Lag7 (16, 17), and coupled with findings from our initial exploratory analysis of the database, an exploratory analysis with an 7-day lag (Lag7) was performed to estimate the pattern of asthma visits in relation to NO_2_ concentration. The effect estimates were presented as odds ratios (*OR*s), OR for childhood acute asthma exacerbation visits per 1 μg/m^3^ increase in NO_2_ concentration, with corresponding 95% confidence intervals (*CI*s). To examine the dose–response relationship, we fitted restricted cubic splines based on a conditional logistic regression model.

In addition, we performed subgroup analyses by sex (male and female) and age (0–3, 4–6, 7–9 years and 10–19 years) based on the demographic characteristics of the patients. Considering the triggers of asthma onset and the sensitivity of the child and adolescent population to environmental exposure factors, we next explored the modification effects of season and AT. First, we calculated separately the asthma visits of children and adolescents during the 24 solar terms (Supplementary Figure S1 illustrates the Gregorian calendar dates corresponding to each of the 24 STs) and initially explored the seasons in which the number of asthma visits changed substantially. Subsequently, we further assessed the effects on asthma visits NO_2_ during summer/autumn and winter/spring. The present work also effect of the assessment of the interaction of NO_2_ and AT on the effect of asthma visits in children and adolescents.

### 2.6 Sensitivity analysis

Multiple sensitivity analyses were performed to evaluate the reliability of our primary results. We adjusted the primary model by incorporating other air pollutants respectively, and individually analyzed the impact of NO_2_ exposure on pediatric asthma visits during 2020–2022 considering that the composition of disease visits may have changed during the COVID-19 pandemic.

All analyses were conducted in R software (version 4.2.0; RStudio Inc.; the USA) with “season” packages. Statistical significance was considered at a *p*-value < 0.05.

## 3 Results

### 3.1 Characteristics of study population and environmental factor

From January 1, 2018 to December 31, 2022, a total of 29, 906 asthma visits, including 27, 636 outpatient visits and 2, 270 inpatient visits, were recorded. Male patients accounted for 19, 508 visits, and female patients accounted for 10,398 visits. Visits were distributed across various age groups as follows: 0*–*3 years (6, 482 visits, 6,200 cases aged 1*–*3 years), 4*–*6 years (13, 465 visits), 7*–*9 years (6, 364 visits) and 10*–*19 years (3, 595 visits). The NO₂ concentration during the same period had a median of 33 μg/m^3^, with the specific median values of other air pollutants and meteorological factors provided in detail in Table 1.

**Table 1:** Summary statistics of visits and environmental factors in children and adolescents with asthma.

Variables	*n* (%)	Min	P_25_	M	P_75_	Max
Asthma visits						
Total	29,906 (100)	0	6	14	24	62
Outpatient	27,636 (92.4)	0	5	13	22	57
Admission	2,270 (7.6)	0	0	1	2	9
Visits classified by gender						
Male	19,508 (65.2)	0	4	9	16	43
Female	10,398 (34.8)	0	2	5	8	26
Visits classified by age						
0–3 years	6,482 (21.7)	0	1	3	5	17
4–6 years	13,465 (45.0)	0	2	6	11	38
7–9 years	6,364 (21.3)	0	1	3	5	20
10–19 years	3,595 (12.0)	0	0	1	3	21
Air pollution (μg/m^3^)						
NO_2_	1826 (−)	9	24	33	46	99
PM_2.5_	1826 (−)	3	26	38	57	239
PM_10_	1826 (−)	5	60	84	119	798
SO_2_	1826 (−)	5	9	12	17	64
CO	1826 (−)	277	618	761	959	3,562
O_3_	1826 (−)	5	62	98	144	282
Meteorological factors						
AT (°C)	1826 (−)	−14.4	2.3	13.4	25.0	37.5
Tmean (°C)	1826 (−)	−9.4	6.0	16.2	24.6	34.3
WS (m/s)	1826 (−)	0.4	1.6	2.0	2.7	7.8
RH (%)	1826 (−)	15	40	55	69	98
Press (hPa)	1826 (−)	975.1	988.0	997.1	1004.3	1021.6

AT = apparent temperature; Tmean = mean temperature; WS = wind speed; RH = relative humidity; Press = pressure.

### 3.2 Correlation analysis

**Figure 1:**
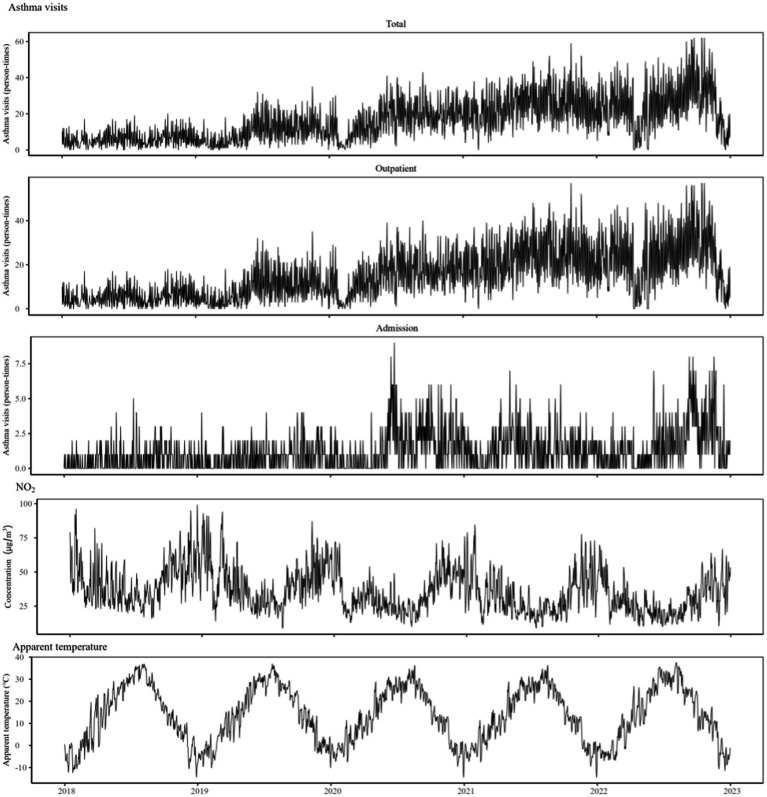
Time trend plots of changes in the asthma visits of children and adolescents and environmental factors.

The time trend plots revealed certain regular and fluctuating trend the changes of asthma visits in children and adolescents, NO_2_ concentration and AT. Especially the changes in NO_2_ concentration and volume of visit showed evident seasonal changes during the study period. A correlation possibly existed between them (Figure 1). Meanwhile, Spearman correlation analysis of air pollutants and meteorological factors unveiled that NO_2_ was positively correlated withPM_2.5_, PM_10_, SO_2_, CO and daily mean pressure and negatively correlated with O_3_, daily mean temperature, RH and WS. In particular, the correlation value of PM_2.5_ with PM_10_ and CO was greater than 0.7, which implies that sensitivity analysis excluded in the model (Supplementary Figure S2).

### 3.3 Effect of NO2 on asthma visits

**Figure 2:**
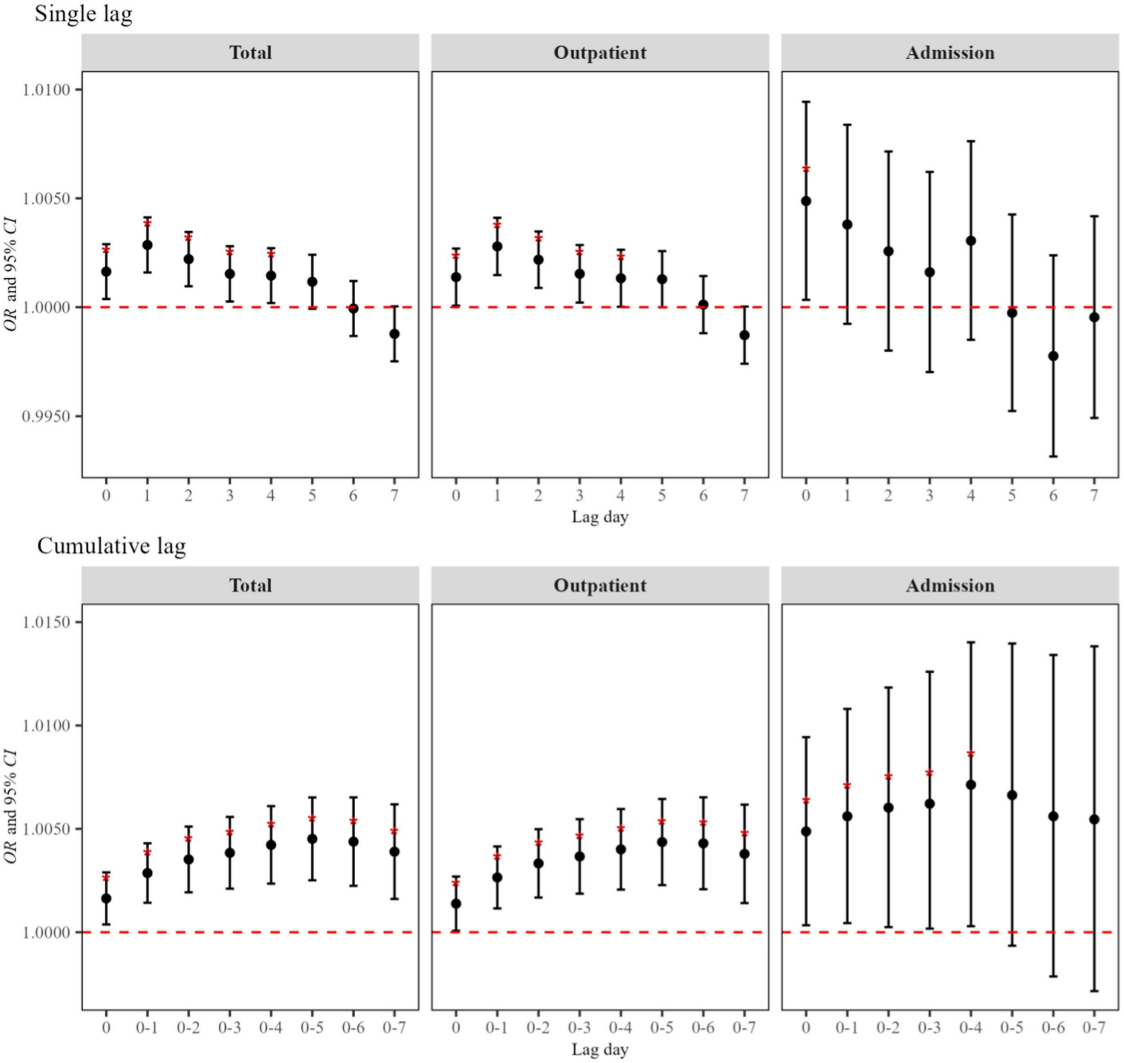
Effects of asthma visits in children and adolescents induced by atmospheric NO_2_ exposure. In the model, the dependent variable is asthma visits, the exposure variable is NO_2_ concentration, and the covariates are apparent temperature and holidays. Single lag effects were estimated for each of the 7 days prior to the visit, while cumulative lag effects were estimated for moving averages over consecutive days during the 7-day period.

**Figure 3:**
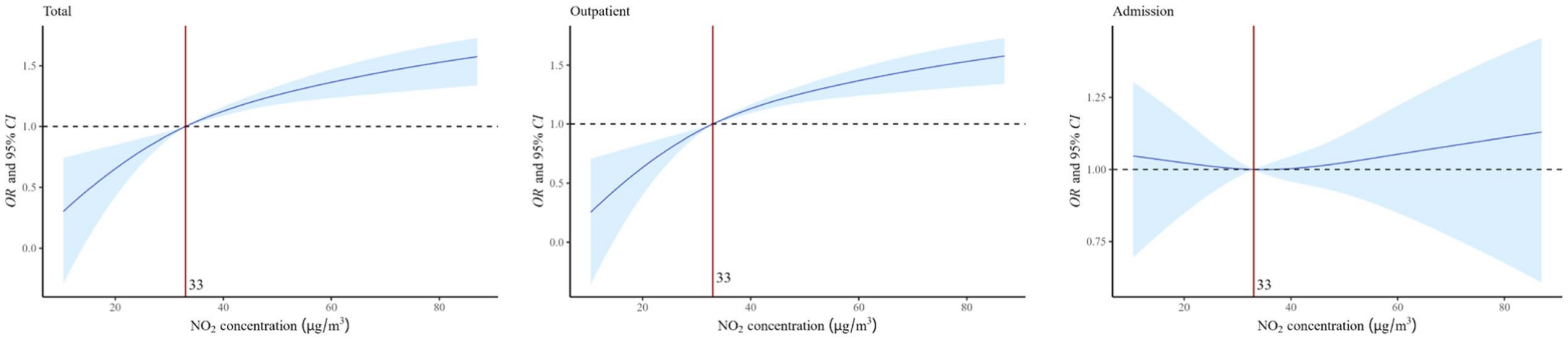
The dose-response relationship of NO₂ exposure and the risk of pediatric asthma visits. In the model, the dependent variable is asthma visits, the exposure variable is NO_2_ concentration, and the covariates are apparent temperature and holidays.

Figure 2 shows the single-day and cumulative lags of atmospheric NO_2_ exposure on asthma visits in children and adolescents. In Lag0–Lag4 and Lag01–Lag07, we observed the notable effects of NO_2_ exposure on the total visits and the strongest effect period at Lag05 (*OR* = 1.005, 95%*CI*: 1.003, 1.007). In addition, significant effects were identified for outpatient and admission visits, with the strongest effects period occurring at Lag05 (*OR* = 1.004, 95%*CI*: 1.002, 1.006) and Lag04 (*OR* = 1.007, 95%*CI*: 1.001, 1.014). The results of the dose–response relationship are presented in Figure 3. The association between NO₂ exposure and the risk of pediatric asthma visits exhibited an approximately parabolic shape, with the nadir of risk at a NO₂ concentration of 33 μg/m^3^. However, the dose–response relationship between NO₂ exposure and the risk of pediatric asthma hospitalization was not statistically significant.

### 3.4 Stratified analysis

**Figure 4:**
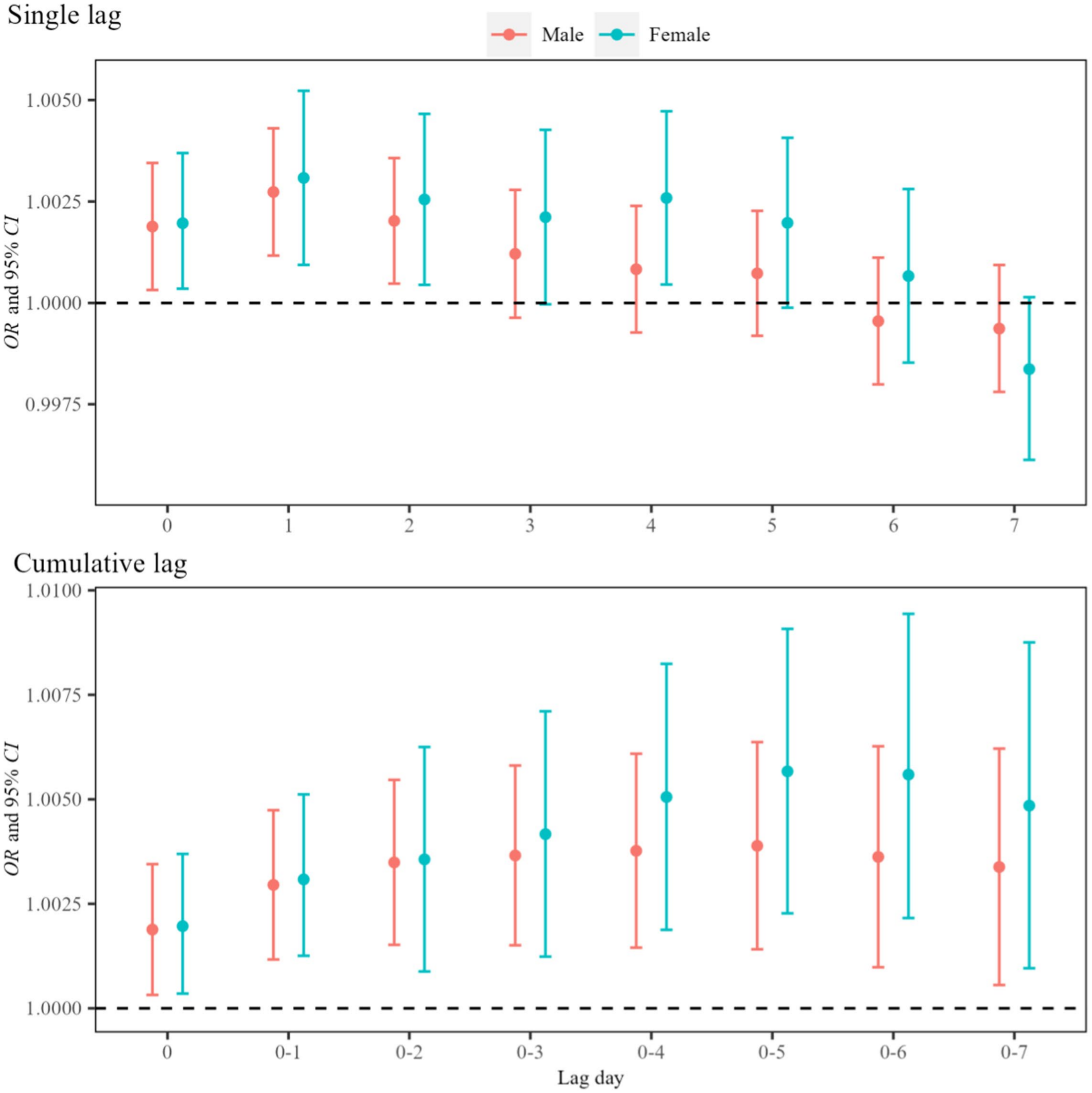
Effects of asthma visits on children and adolescents of different gender induced by atmospheric NO_2_ exposure. In the model, the dependent variable is asthma visits, the exposure variable is NO_2_ concentration, and the covariates are apparent temperature and holidays.

**Figure 5:**
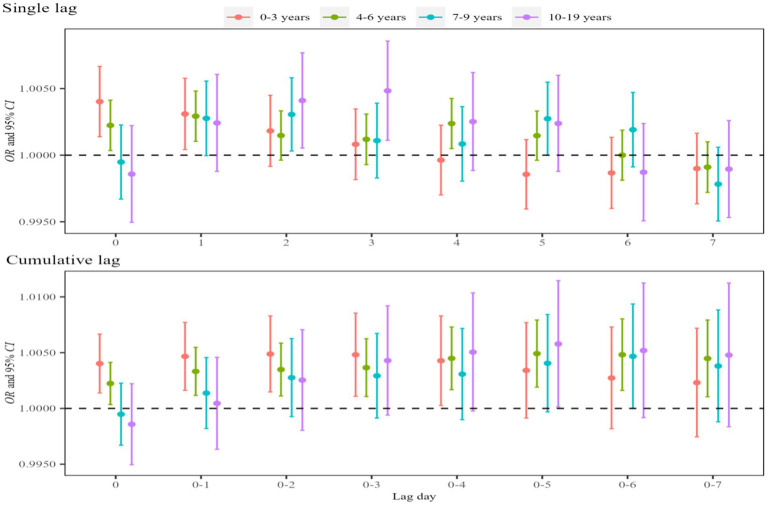
Effects of asthma visits on children and adolescents of different age groups induced by atmospheric NO_2_ exposure. In the model, the dependent variable is asthma visits, the exposure variable is NO_2_ concentration, and the covariates are apparent temperature and holidays.

Figure 4 shows that atmospheric NO_2_ exposure significantly influenced asthma visits among male and female children and adolescents and then reached the maximum on Lag05. The effect estimate was higher in females (*OR* = 1.006, 95%*CI*: 1.002, 1.009) compared with males (*OR* = 1.004, 95%*CI*: 1.001, 1.006). Age-stratified analysis revealed statistically significant effects for all age groups, with the strongest effects observed in adolescents aged 10–19 years (*OR* = 1.005, 95%*CI*: 1.001, 1.009), followed by those aged 0–3 years (*OR* = 1.004, 95%*CI*: 1.001, 1.007), 4–6 years (*OR* = 1.003, 95%*CI*: 1.001, 1.005), and 7–9 years (*OR* = 1.003, 95%*CI*: 1.001, 1.006) and reached the maximum on Lag3, Lag0, Lag1 and Lag2 for these groups, respectively (Figure 5). The corresponding *p*-values for the effect estimates (*OR* and 95%*CI*) are provided in Supplementary Table S1.

### 3.5 Modification effects analyses

#### 3.5.1 Effects of 24 solar terms and seasons

**Figure 6:**
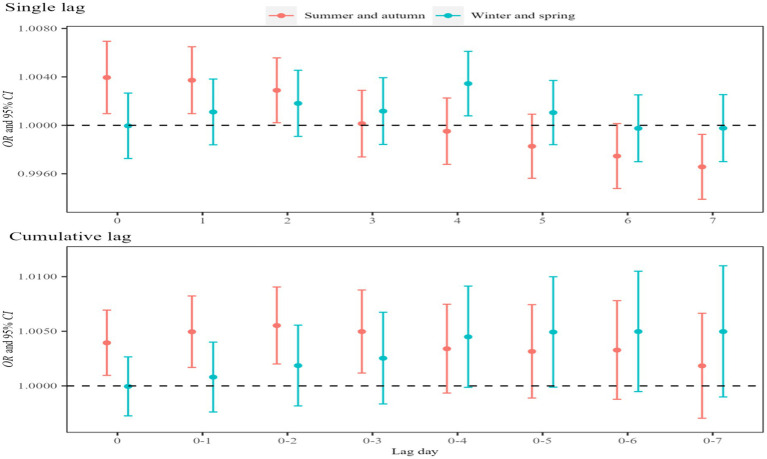
Effects of atmospheric NO_2_ exposure on children and adolescents in summer–autumn and winter–spring seasons. In the model, the dependent variable is asthma visits, the exposure variable is NO_2_ concentration, and the covariates are apparent temperature and holidays.

The asthma-related visits among children and adolescents varied dramatically across the 24 STs. The White Dews (one of the 24 Chinese solar terms, falls in early September) had the largest visits (1,646 visits, 5.50%), and the Spring Begins (one of the 24 Chinese solar terms, falls in early February) had the lowest cases (781 visits, 2.61%). Notably, marked changes in asthma visits were noted during the transitions between spring–summer and autumn–winter seasons (Supplementary Figure S1). Further analysis revealed the higher effect of atmospheric NO₂ exposure on asthma visits during the summer–autumn seasons (*OR* = 1.006, 95%*CI*: 1.002, 1.009, Lag02) than during the winter–spring seasons (*OR* = 1.003, 95%*CI*: 1.001, 1.006, Lag04) (Figure 6; Supplementary Figure S1).

#### 3.5.2 Interaction between NO2 and apparent temperature

**Figure 7:**
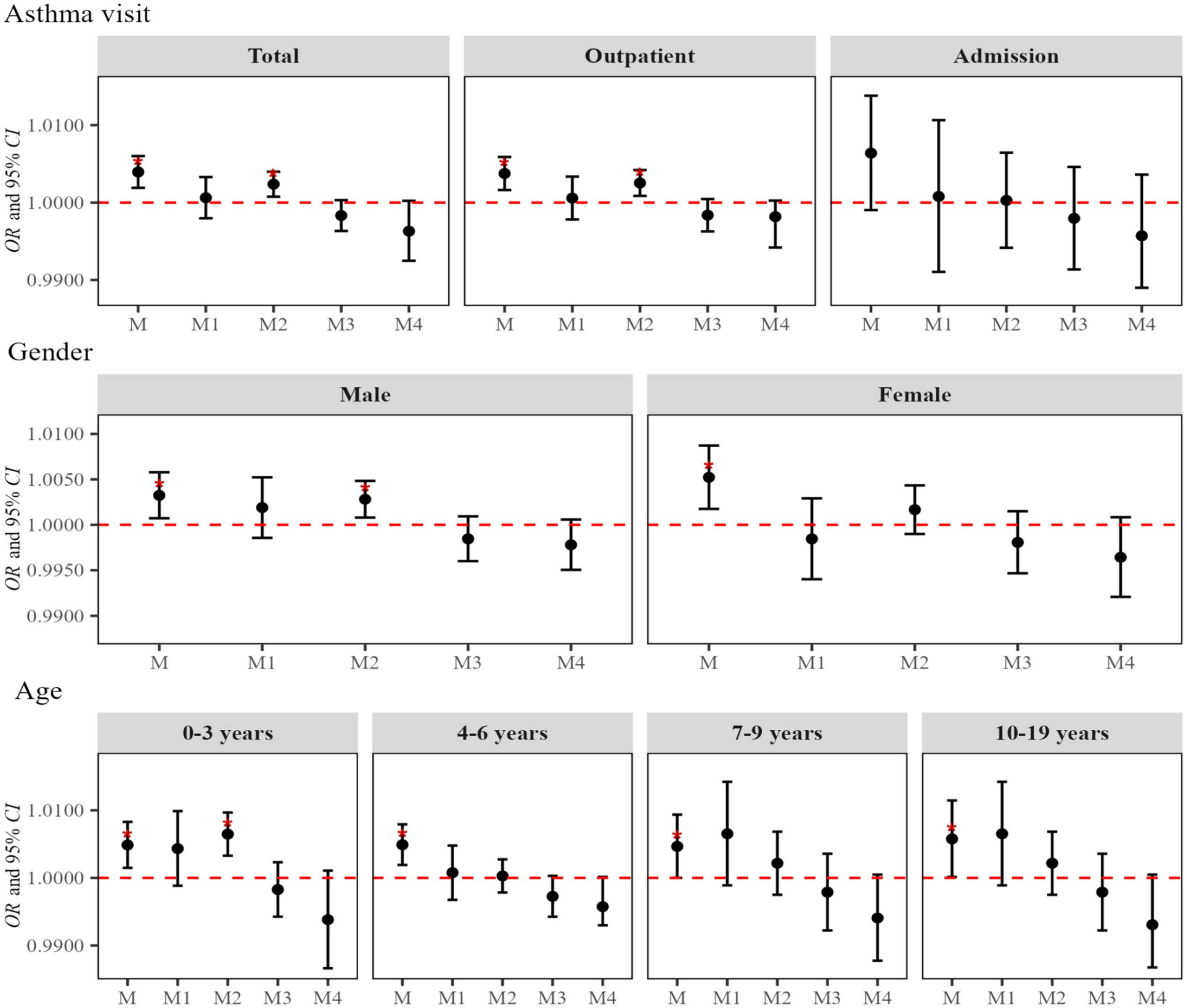
Interaction between atmospheric NO_2_ and AT on asthma visits among children and adolescents. In the model, the dependent variable is asthma visits, the exposure variable is NO_2_ concentration, and the covariates are apparent temperature and holidays. With moderate AT used as a reference, M denotes the independent effects of atmospheric NO_2_, M1 refers to the interaction between NO_2_ and extremely low AT, M2 indicates the interaction between NO_2_ and low AT, M3 represents the interaction between NO_2_ and high AT; M4 means the interaction between NO2 and extremely high AT.

Figure 7 illustrates the interaction between atmospheric NO_2_ and AT on asthma visits among children and adolescents. With moderate AT used as the reference, the modification effects of extremely low, low, high and extremely high AT on the effect of atmospheric NO_2_ exposure on asthma visit volumes were estimated. Results reveal that the interaction between atmospheric NO_2_ and low AT was significant among total visits, outpatient visits, male and age 0–3 years, with corresponding effect estimates equal to 1.002 (95%*CI*: 1.001, 1.004), 1.003 (95%*CI*: 1.001, 1.004), 1.003 (95%*CI*: 1.001, 1.005) and 1.006 (95%*CI*: 1.003, 1.010), respectively (*p*-values see Supplementary Table S1).

### 3.6 Sensitivity analysis

During the maximum exposure period identified by the main model, dual- and multi-pollutant models were fitted, and COVID-19 pandemic period were individually analyzed for sensitivity analysis. The effects of atmospheric NO₂ on asthma visits remained mostly unchanged, except for inpatient visits, which indicates the robustness of the models used in this research (Supplementary Figure S3).

## 4 Discussion

Air pollution and meteorological factors exert varying degrees of influence on the emergence and development of a number of respiratory diseases, especially asthma attacks in children (5, 18). Under the background of abnormal global climate change, scholars should focus on the dual challenge of priority pollutants and meteorological factors on asthma attacks in children.

This study found that the male-to-female ratio of pediatric asthma visits was approximately 2:1. This gender distribution is highly consistent with our previously published studies based on the same hospital setting, which have, respectively, focused on 66.37% vs. 33.62 and 66.2% vs. 33.8% (19, 20). Moreover, this ratio is comparable to the data from another medical institution, which documented a male-to-female ratio as 68.64% vs. 31.36% in its investigation of childhood asthma (21). These findings collectively suggest that the observed gender imbalance may reflect the actual epidemiological characteristics of childhood asthma in this regional population. There was an apparent positive trend in asthma visits over the study period (2018–2022). The increasing trend in asthma visits may be attributed to the rising prevalence of childhood asthma, which is supported by the Global Burden of Disease (GBD) data and relevant literature (22).

In this study, the daily average NO₂ concentration during the study period was 33 μg/m^3^, which is substantially lower than the Grade II standard limit (80 μg/m^3^) specified in the Chinese Ambient Air Quality Standards (GB 3095–2012) (23), indicating that atmospheric NO₂ concentrations complied with the national standard. However, further analysis of the impact of NO₂ exposure on asthma visits revealed that NO₂ exposure significantly increased the risk of asthma visits among children and adolescents. This is generally consistent with existing studies. Dong et al. reported that a 10 μg/m^3^ increase in NO₂ concentration in Northeast China led to a 26% rise in asthma prevalence (24). Similarly, Liu et al. demonstrated a significant association between elevated NO₂ concentrations and increased asthma cases among children aged 6–13 years (25). Toxicological evidence indicates that NO₂ toxicity is related to lipid peroxidation in cell membranes, with a high NO₂ exposure inducing small airway permeability, airway inflammation and related effects (26, 27). More importantly, the exposure-response analysis conducted in this study further revealed that when the NO₂ concentration exceeded 33 μg/m^3^, the risk of pediatric asthma visits became statistically significant. This is inconsistent with the NO₂ exposure threshold for the lowest risk of pediatric asthma visits identified in studies conducted in Wuxi and California (28, 29). This may be attributed to regional differences in meteorological factors.

The results of the stratified analysis and effect modification analysis are as follows. This study revealed sex- and age-specific disparities in the effect of atmospheric NO₂ on pediatric asthma visits, with a higher number of females than males and more children belonging to 10–19 and 0–3 years age groups than other age-groups. Studies have reported higher effects in females compared with males, consistent with our research (30). However, the age group differences observed in this study were primarily based on the following considerations. Individuals aged 10–19 engage in notably more outdoor activities and display weaker self-awareness of health protection, which leads to a greater exposure to NO_2_ (31). Meanwhile, children aged 0–3 are in a critical stage of rapid lung development, which make them susceptible to NO_2_ exposure (32). In addition, this study, based on the analysis of visit patterns across the 24 STs, revealed marked changes in asthma visits during transitions between spring–summer and autumn–winter seasons. Further analysis revealed the higher effect of atmospheric NO₂ exposure on asthma visits during the summer–autumn seasons than during the winter–spring seasons. The main consideration is that asthma, as a chronic disease, does not affect children’s daily life and study when not exacerbated. Therefore, children with asthma usually seek medical treatment during the summer vacation when they have sufficient time and convenient travel. But, effect modification analysis revealed that only the low AT and NO_2_ exposure exhibited a statistically significant interaction effect on pediatric asthma visits, with a moderate AT serving as the reference. Low temperatures often coincide with high air pollution concentrations, which carry oxidative components that induce reactive oxygen species production in the lungs, deplete antioxidants and disrupt immune function; such conditions result in allergic responses (33). However, with the dissemination of public health knowledge regarding the adverse effects of low temperatures, parents have adopted corresponding protective measures for their children during extremely cold weather to reduce exposure opportunities. Therefore, no statistically significant interaction effect on pediatric asthma visits was identified between extremely low AT and NO_2_ exposure.

However, this study has some limtations and caution should be exercised when generalizing the findings. Firstly, while the case-crossover design study uses case themselves controls, mitigating the influence of most individual factors on study outcomes, it may not fully account for as yet unidentified or unadjusted confounders inherent in real-world scenarios. Secondly, ambient air pollutant concentrations were assessed at a relatively coarse urban scale, which may limit the representativeness of exposure estimates. Thirdly, the study could not accounting for variations in children’s living environments and activities during winter and summer vacations, viral respiratory epidemics and seasonal allergens (pollen counts). Lastly, this study treats Jinan City as representative and the data were obtained from one hospital, potential selection bias and limited representativeness limiting the generalizability of our results. Outcomes may deviate for cities with differing geographical locations and climate patterns. Future multi-center and large-sample prospective studies are warranted to improve representativeness and generalizability.

## 5 Conclusion

In conclusion, atmospheric NO_2_ significantly increased asthma visits among children and adolescents, with seasons and AT having modifying effects. Female and 10–19 years old children and adolescents were vulnerable populations. These findings highlight the urgent need for increased awareness and action from relevant agencies. Relevant departments continue to strengthen the management of atmospheric NO_2_ under the guidance of the *National Three-Year Action Plan for Winning the Battle for the Blue Sky* and the *Jinan Air Pollution Action Plan (Phase III)*. During transitions between spring–summer and autumn–winter seasons, the dissemination of scientific knowledge regarding meteorological factors and the asthma implications of NO_2_ pollution must be prioritized. Alternatively, children and adolescents with asthma are reminded to reduce outdoor activities when the Air Quality Forecast Information Dissemination System indicates a sudden increase in NO_2_ concentrations.

## Data Availability

The datasets presented in this article are not readily available because this dataset may not be used beyond the designated research domains, and requires written authorization from the original authors. Requests to access the datasets should be directed to wxl10188@126.com.
