# Supplementary material for: Impact of atmospheric NO2 on pediatric asthma visits in Jinan: effect modification by season and apparent temperature
Source: Front Public Health. 2026 May 8;14:1793681. doi: 10.3389/fpubh.2026.1793681 (PMC13195748; doi:10.3389/fpubh.2026.1793681)
Supplement: Supplementary file 1 [file Image_1.pdf]

Supplemental Materials

## 2 Materials and methods

### 2.3 Data collection on air pollutions and meteorological variables

Quality control for air pollutant data involved: calculating daily means as arithmetic averages from 33 monitoring stations across Jinan; requiring at least 20 of 24 hours (or 6 of 8 hours) of data for a valid day, otherwise treating as missing; excluding stations with >25% missing data; and imputing remaining missing values with adjacent averages. Outliers were selectively removed, and data were validated for logical consistency.

### 2.5 Statistical analysis

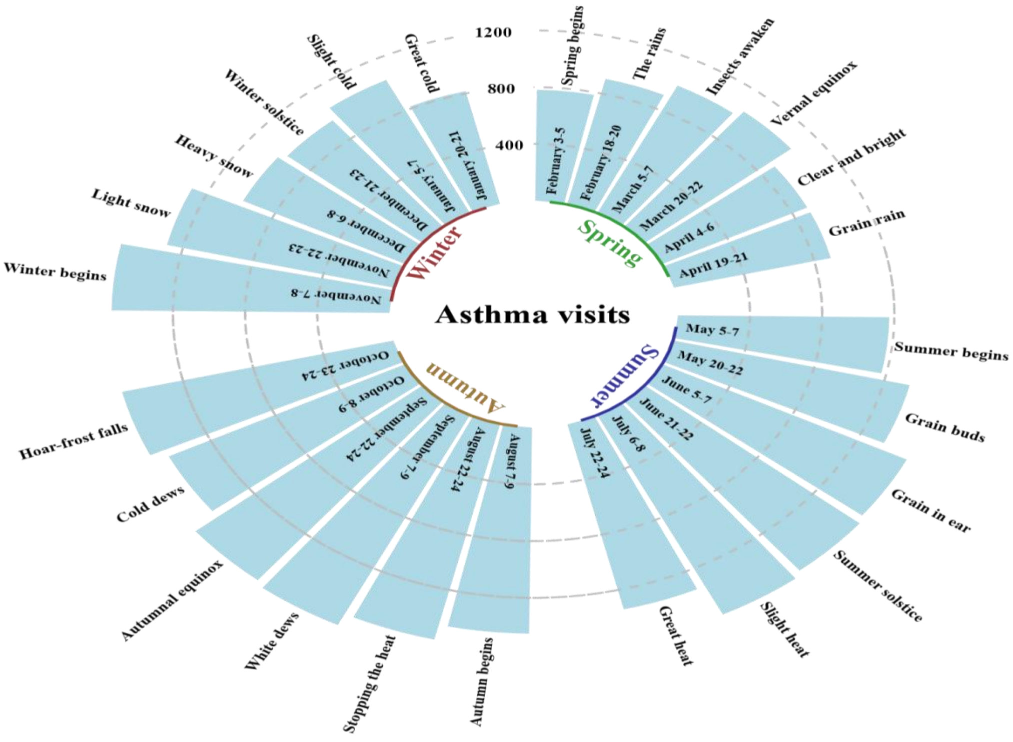

Supplementary Figure S1  Asthma visits among children and adolescents throughout the 24 solar terms

## 3 Results

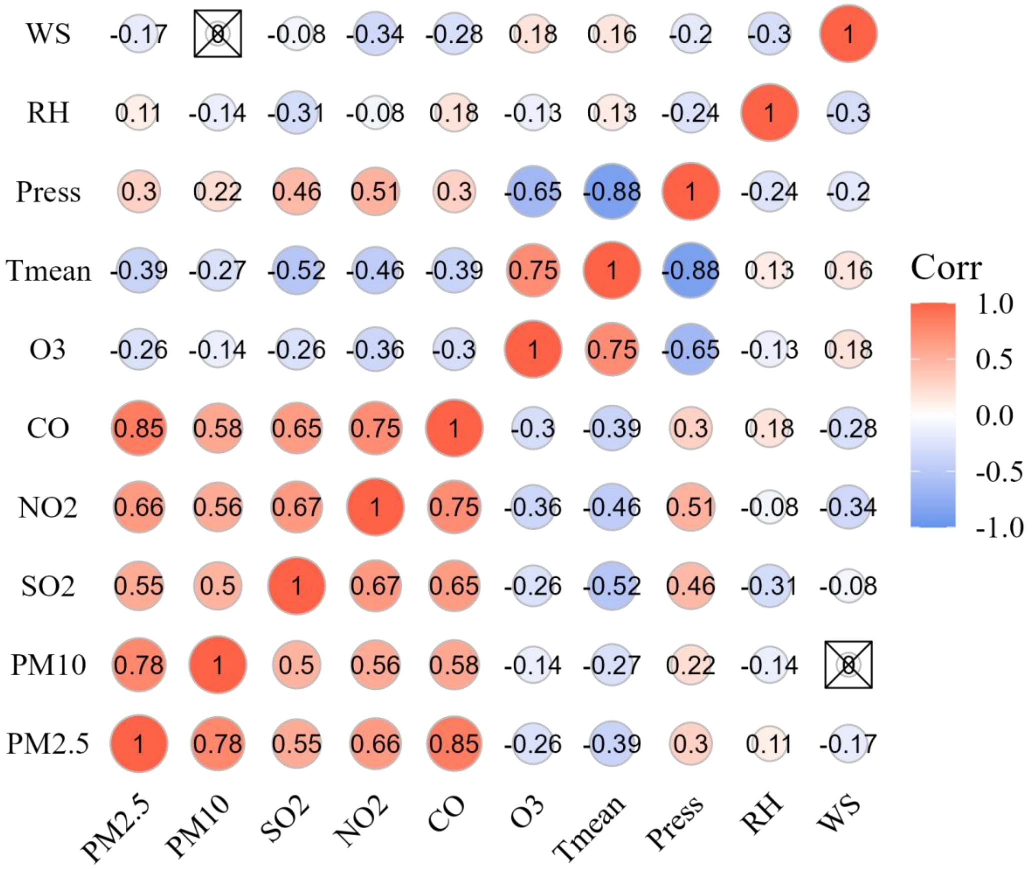


Supplementary Figure S2  Matrix plot of the Spearman correlation coefficient between atmospheric pollutant concentrations and meteorological factors

Supplementary Table S1  The *OR*s and *P*-values for the corresponding results of the stratified analysis

| Variable | *OR* (95%CI) | *P* |
|---|---|---|
| Gender | | |
| Male | 1.004 (1.001, 1.006) | 0.002 |
| Female | 1.006 (1.002, 1.009) | 0.001 |
| Age | | |

| | | |
|---|---|---|
| 0-3 years | 1.004 (1.001, 1.007) | 0.003 |
| 4-6 years | 1.003 (1.001, 1.005) | 0.002 |
| 7-9 years | 1.003 (1.001, 1.006) | 0.029 |
| 10-19 years | 1.005 (1.001, 1.009) | 0.011 |
| Season | | |
| Summer and autumn | 1.006 (1.002, 1.009) | 0.002 |
| Winter and spring | 1.003 (1.001, 1.006) | 0.011 |
| Modification of AT | | |
| Total visits | 1.002 (1.001, 1.004) | 0.004 |
| Outpatient | 1.003 (1.001, 1.004) | 0.003 |
| Male | 1.003 (1.001, 1.005) | 0.006 |
| 0-3 years | 1.006 (1.003, 1.010) | 0.001 |

Abbreviations: AT=apparent temperature

Note: P ≤ 0.05 was considered statistically significant.

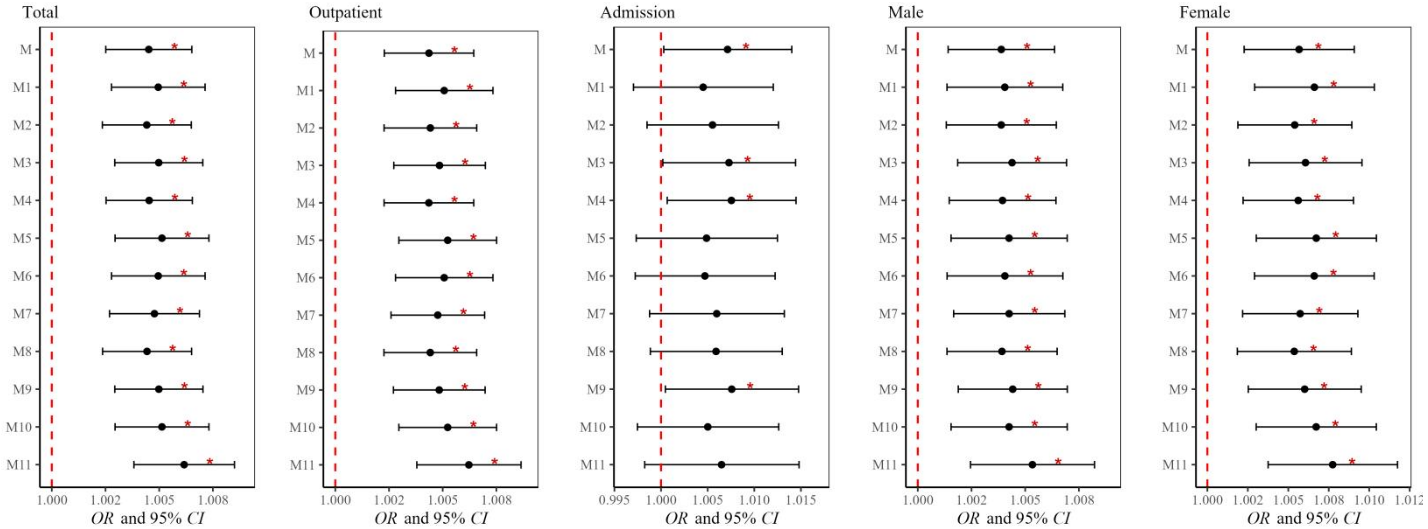

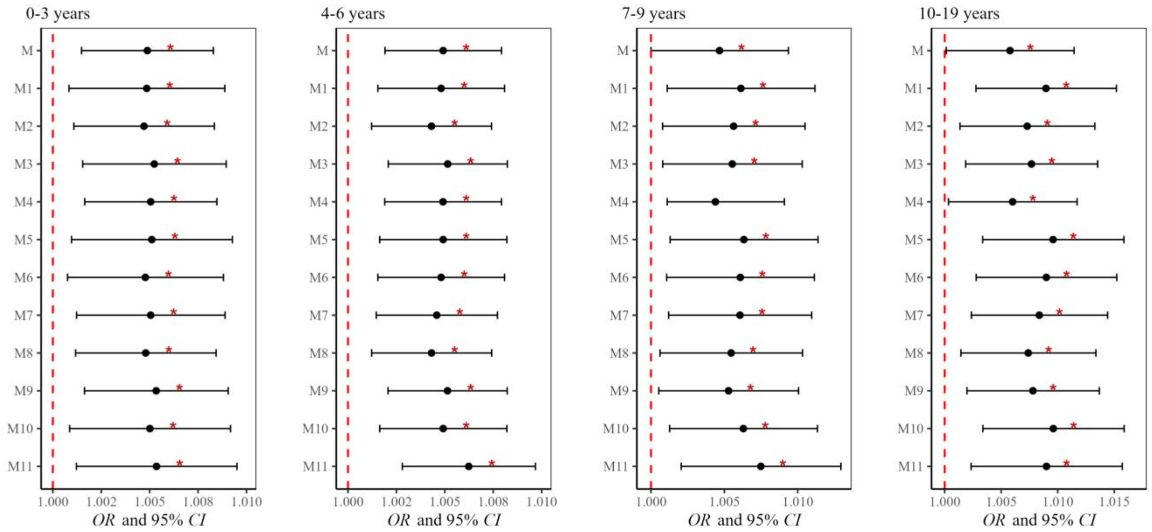


Supplementary Figure S3 Sensitivity analysis of effects on asthma visits in children and adolescents induced by atmospheric $NO_2$

Note: M was the main model; M1 for adjustment of $PM_{2.5}$; M2 for adjustment of $PM_{10}$; M3 for adjustment of $SO_2$; M4 for adjustment of $O_3$; M5 for adjustment of $PM_{2.5}$ and $SO_2$; M6 for adjustment of $PM_{2.5}$ and $O_3$; M7 for adjustment of $PM_{10}$ and $SO_2$; M8 for adjustment of $PM_{10}$ and $O_3$; M9 for adjustment of $SO_2$ and $O_3$; M10 for adjustment of $PM_{2.5}$, $SO_2$ and $O_3$； M11 for COVID-19 pandemic period.
